# Dissecting the mechanisms of pathogenesis in cerebral malaria

**DOI:** 10.1371/journal.ppat.1010919

**Published:** 2022-11-17

**Authors:** Arathy Ramachandran, Amit Sharma

**Affiliations:** Molecular Medicine Group, International Centre for Genetic Engineering and Biotechnology, New Delhi, India; University of Wisconsin Medical School, UNITED STATES

## Abstract

Cerebral malaria (CM) is one of the leading causes of death due to malaria. It is characterised by coma, presence of asexual parasites in blood smear, and absence of any other reason that can cause encephalopathy. The fatality rate for CM is high, and those who survive CM often experience long-term sequelae, including cognitive and motor dysfunctions. It is unclear how parasites sequestered in the lumen of endothelial cells of the blood–brain barrier (BBB), and localised breakdown of BBB can manifest gross physiological changes across the brain. The pathological changes associated with CM are mainly due to the dysregulation of inflammatory and coagulation pathways. Other factors like host and parasite genetics, transmission intensity, and the host’s immune status are likely to play a role in the development and progression of CM. This work focuses on the pathological mechanisms underlying CM. Insights from humans, mice, and in vitro studies have been summarised to present a cohesive understanding of molecular mechanisms involved in CM pathology.

## Introduction

Malaria is a major vector-borne disease endemic to tropical regions in central and south America, Asia, Africa, eastern Europe, and the South Pacific, with the highest transmission found in sub-Saharan Africa. The most common causative agents are *Plasmodium falciparum* and *Plasmodium vivax*. The infection is spread to the human hosts by female *Anopheles* mosquitoes. The severity of malaria ranges from asymptomatic to mild and uncomplicated to severe and complicated. Human cerebral malaria (CM) is one of the severe complications associated with malaria [1]. Other complications include respiratory distress, metabolic acidosis, severe malaria anaemia (SMA), and hyperlactatemia. The World Health Organization (WHO) outlined 3 criteria for diagnosis of CM: the presence of asexual parasites in the peripheral blood smear, unrousable coma, and exclusion of any other known causes of encephalopathies [2].

The initial symptoms that the patients typically present with are high fever and neurological symptoms such as altered levels of consciousness and/or coma. Other symptoms, which have been reported only in a subset of patients, are muscle pain, abnormal rigidity in postures involving arms, legs, and neck, and convulsions [3]. Primary systemic changes common across patients are hyperpyrexia, hypoglycemia, electrolyte imbalance, metabolic acidosis, SMA, and acute kidney injury. The symptoms progress differently between children and adults. In paediatric cases, the coma develops rapidly and lasts for a shorter duration, and convulsions and brainstem abnormalities are more common. Whereas, in adults, coma develops gradually with increasing drowsiness and confusion but lasts longer. Also, seizures are less common in adults, while mortality due to CM is higher [4]. In high transmission areas, CM is more common in children under 5 years of age than in adults, and in low transmission areas, it is more common in older children or adults. It is unclear why the symptoms are so diverse and manifest in only some patients. In addition to the heterogeneous nature of the symptoms, what makes it challenging for clinicians to treat CM is that more than 90% of the total CM cases are reported in sub-Saharan Africa in resource-poor settings [5]. Without treatment, CM is fatal [6]. The mortality associated with paediatric CM was estimated to be 18% in the artesunate arm of AQUAMAT trial, while it was estimated to be 30% in adults in the artesunate arm of SEAQUAMAT trial [4]. Oluwayemi and colleagues reported that the prevalence of neurological sequelae in their study cohort of 160 children was as high as 13.7% at the time of discharge [7].

The lack of early diagnosis and more effective treatment strategies makes it evident that there is a lacuna in our understanding of the pathological mechanisms underlying the development of CM. This work integrates results from epidemiological, postmortem, and mouse model studies to present a comprehensive summary of what we understand about CM pathogenesis.

**Fig 1:**
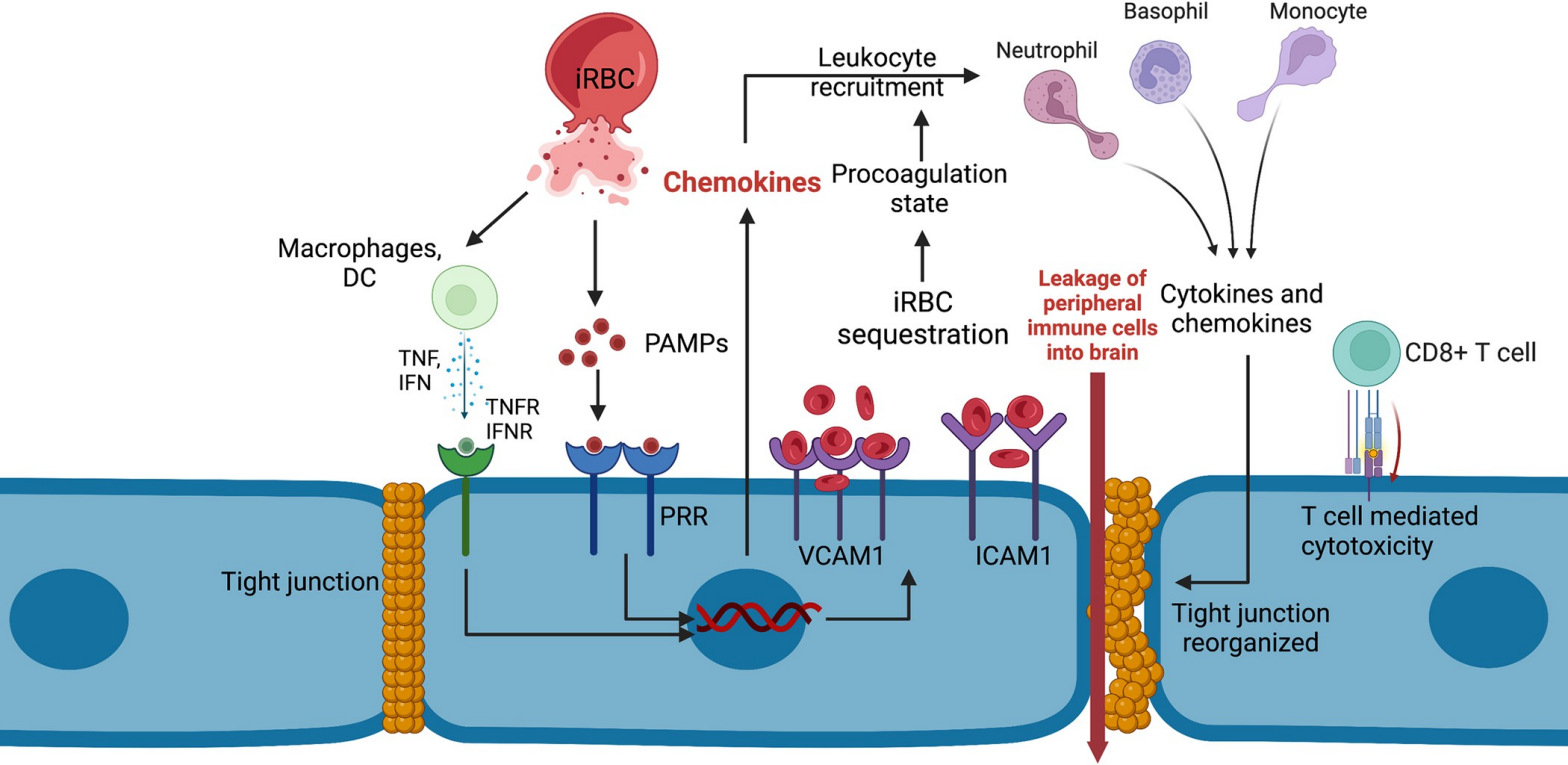
Pathogenic mechanisms leading to the breakdown of BBB. Rupture of iRBCs and release of merozoites into the bloodstream activate the macrophages and dendritic cells. This results in elevation of serum levels of TNF and IFN gamma. TNF and IFN gamma are known to up-regulate the expression of VCAM1 and ICAM1 on the endothelial cells. This in turn leads to an increase in the recruitment of iRBCs to the endothelial cells, thus promoting localised inflammation and procoagulation pathways. Meanwhile, the PRRs, such as Toll-like receptors expressed on the endothelial cells, recognise malarial PAMPs and trigger the secretion of proinflammatory cytokines like IL-6, IL-12, and TNF. The cytokines and chemokines released locally recruit leukocytes such as basophils, neutrophils, and natural killer cells to the site of inflammation. These cells, upon activation, release chemokines like MIP-1α and MIP-1β, which recruit more leukocytes to the site of inflammation. The elevated levels of chemokines and cytokines also induce reorganisation of tight junction proteins affecting the BBB integrity. Endothelial cells also present the parasitic antigen via MHC Class I molecules to CD8+ T cells in the brain. This triggers the CD8+ T cell–mediated cell death of endothelial cells mediated by granzyme B. These molecular events in tandem disrupt the BBB integrity and contribute to CM pathogenesis. This figure was made using BioRender.

**Fig 2:**
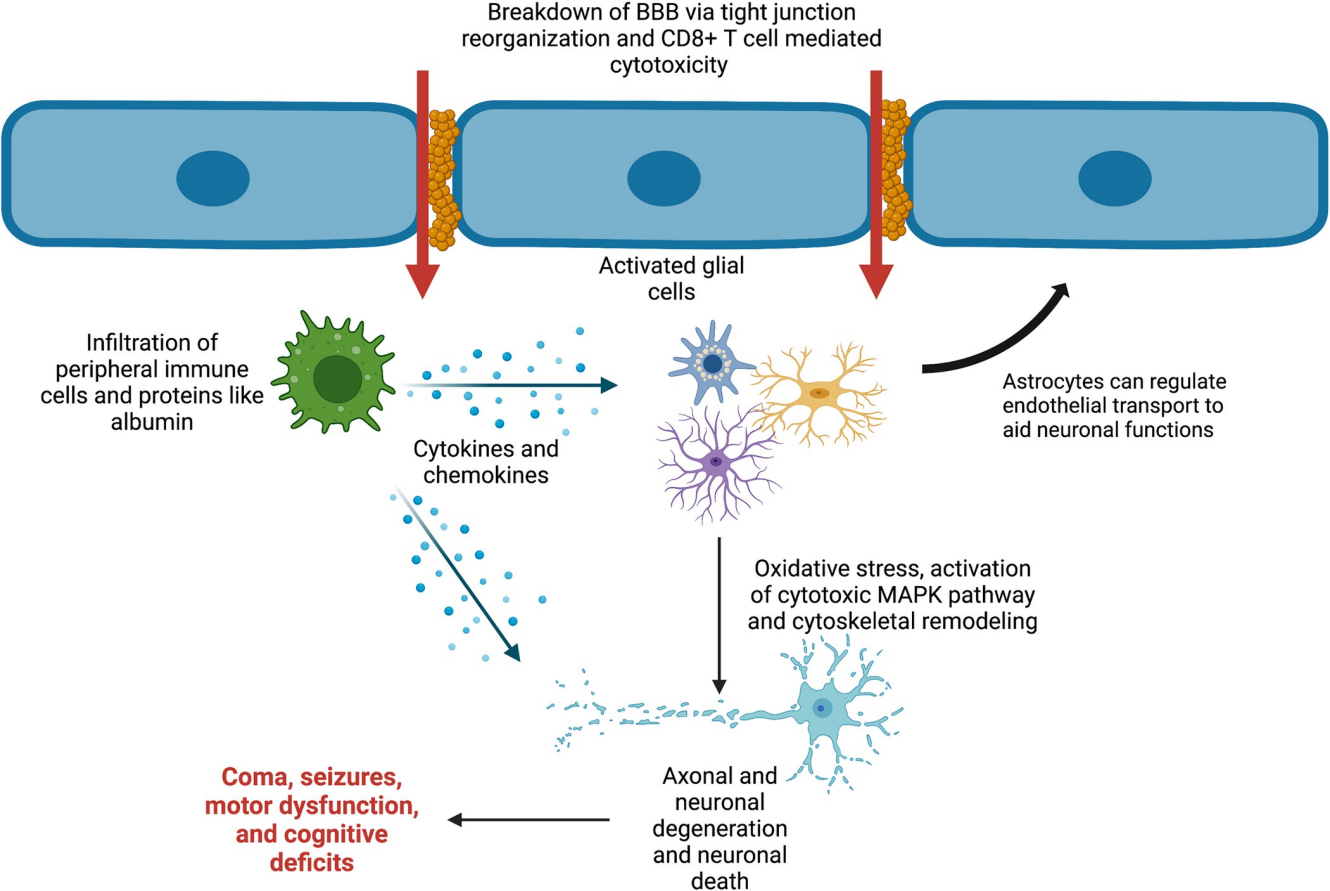
Pathogenic changes in the brain following the breakdown of BBB. The breakdown of endothelium results in vascular leakage of parasite-derived proteins and peripheral immune cells such as neutrophils into the brain. These changes trigger the activation of astrocytes and microglia and increase proinflammatory cytokines and chemokines in the brain. Glial activation and changes in inflammation status contribute to oxidative stress and up-regulation of MAP kinase-mediated cytotoxic pathways. Oxidative stress can affect actin polymerisation via calpain 1 and calpain 2, which might contribute to axonal loss. The breakdown of BBB also leads to an increase in albumin levels and loss of proton pumps in the brain, which might be one of the reasons for cerebral oedema. Together, these events lead to neuronal death, resulting in coma along with cognitive and/or and motor disabilities. This figure was made using BioRender.

Our discussion is divided into 2 parts: (**i) the pathological changes that occur in the brain vasculature** following sequestration of infected erythrocytes in the endothelium of the blood–brain barrier (BBB) (Fig 1); and (**ii) the changes that occur in the brain following the endothelial damage** (Fig 2). Notable progress has been made to further our knowledge of how CM develops, but there are significant gaps in our understanding of this disease. A deeper understanding of the physiological processes underlying CM will help us identify new therapeutic targets and to possibly design more efficient treatment strategies. This will significantly reduce the number of deaths or morbidity caused by CM.

### i) Pathological changes in brain vasculature

Postmortem studies on the brains of adult CM patients showed haemorrhagic lesions in the cerebral white matter and corpus callosum, inflammation in basal ganglia, brainstem, corpus callosum, thalamus and cerebellum, axonal injury, localised disintegration of BBB, and loss of myelin sheath [8]. Whereas, in juvenile patients, postmortem analysis of brains revealed changes such as mild to moderate increase in brain weight, disruption of BBB, presence of T cells and macrophages, ring haemorrhage, and diffused axonal and myelin damage [9]. Two hypotheses emerged in the 1990s to explain the pathological changes associated with CM: *the sequestration hypothesis* and *the cytokine hypothesis* [10,11]. The sequestration hypothesis is based on infected red blood cells’ (iRBCs) ability to sequester in the brain vasculature. This leads to occlusion of the vessels and endothelial breakdown. On the other hand, the inflammation hypothesis suggests that the imbalance in the inflammation status leads to endothelial dysfunction and neuronal injury. Later, as significant discoveries about both CM and normal human physiology were made, a *unified hypothesis* was proposed to explain the CM pathogenesis [12]. By then, a significant number of studies had shown an association between the procoagulant state of the host and CM, owing to the presence of haemorrhages in the brains of CM patients. van der Heyde and colleagues proposed the idea that all the 3 events (sequestration of iRBCs in the vasculature, inflammation, and dysregulation of coagulation pathways) contribute to the pathogenesis of CM and that they are not mutually exclusive. It is now increasingly evident that CM is a complex disorder with a heterogenous clinical manifestation. Here, we have summarised the events leading to endothelial breakdown under 4 sections: (a) *sequestration of iRBCs in the endothelium of BBB*; (b) *endothelial activation and development of a procoagulant state*; (c) *inflammation*; and (d) *endothelial breakdown*.

### a) *Sequestration of iRBCs in the endothelium of BBB*

Endothelial cells are the thick-walled cells that make the single-layered lining of our vasculature. It is widely accepted that the first step in the progression of malaria to CM is iRBC sequestration in the endothelium of BBB. One of the mechanisms mediating the sequestration of iRBCs is the interaction of host cell receptors with *P*. *falciparum* erythrocyte membrane protein 1 (PfEMP1) (reviewed in [13]). PfEMP1 belongs to a protein family encoded by the *var* genes. A parasite genome consists of approximately 60 var genes. However, a single var gene is expressed at a given time in a given cell. Switching var gene expression is one factor that helps the parasite evade the host immune system. PfEMP1 has been shown to interact with a diverse array of host receptors: cluster of differentiation 36 (CD36), intracellular adhesion molecule 1 (ICAM1), P-selectin, cluster of differentiation 31 (CD31), thrombospondin, vascular cell adhesion molecule 1 (VCAM1), E-selectin, heparan sulphate, neural cell adhesion molecule (NCAM), fractalkine, endothelial protein C receptor (EPCR), integrin ανβ3, hyaluronan binding protein 1 (HABP1), and fibronectin. On the BBB specifically, PfEMP1 expressed on the iRBCs has been shown to interact with the endothelial cells via VCAM1, ICAM1, and EPCR [14,15,16]. This immobilises the iRBCs on the endothelial cells and leads to their sequestration on the BBB, resulting in endothelial activation.

### b) *Endothelial activation and development of a procoagulant state*

Endothelial activation is a state characterised by an increase in the number of leukocytes and pro-coagulatory markers in the endothelial cell. One such procoagulatory marker is von Willebrand Factor (vWF). Levels of vWF in malaria patients are directly correlated with the severity of infection [17]. vWF is released into the blood from endothelial cells in an active conformation. It then binds to the platelet receptors such as glycoprotein Ia—V—VI complex. This interaction triggers the intravascular platelet aggregation [18]. Platelet aggregation in brain microvasculature is a common feature of the human CM. Platelets can adhere directly to the endothelial cells via cluster of differentiation 40 and 41 (CD40 and CD41) or to the vWF multimers expressed on the endothelial surface [17,19]. Platelets can also form multimeric structures with iRBCs and endothelial receptors, thus promoting the recruitment of iRBCs to the cerebral vasculature. This multimeric interaction between vWF, iRBCs, endothelial receptors, and platelet aggregates leads to clumping and vascular constriction.

Platelet activation also leads to the secretion of platelet microparticles (PMPs). PMPs are small plasma membrane vesicles ranging in size from 0.1 to 1 micrometre and express a diverse range of receptors on their surface. These include P-selectin, glycoprotein complexes such as IIb/IIIa and Ib/IX, tissue factor (TF), and phosphatidylserine. These receptors further promote platelet-endothelial adhesion and coagulation. Platelet activation also leads to the translocation of alpha granules to the surface of platelets. Alpha granules are secretory organelles present inside the platelets and contain receptors such as P-selectin. Platelet activation leads to the expression of these receptors on the platelet surface, which then promotes endothelial activation and iRBC sequestration.

Conversion of prothrombin into thrombin is an essential step in the coagulation cascade. Thrombin production is inhibited when thrombomodulin interacts with and activates protein C [20]. In vitro studies report that the chondroitin sulphate A side chain of thrombomodulin can interact with receptors on iRBCs [20], suggesting that a low-affinity interaction between thrombomodulin and iRBCs will reduce the availability of thrombomodulin for protein C activation and, hence, down-regulate the anticoagulative pathway. Protein C levels and activity are inversely correlated with severity and parasite density of Pf infection in vivo, suggesting that anticoagulation pathways are down-regulated in severe malaria [21,22]. Also, severe Pf infection is associated with significantly low levels of another anticoagulant protein called antithrombin III [23].

### c) *Inflammation*

A potential inflammatory cascade beginning from the liver stage infection and leading up to the breakdown of BBB was first presented by Dunst and colleagues in their review of cytokines and chemokines associated with CM pathology [24]. The liver stage infection is silent as far as the symptoms are concerned. However, the parasites interact with the innate immune system during this stage, as evidenced by the presence of dendritic cells and macrophages around *Plasmodium berghei–*infected hepatocytes. A spike in interferon-gamma levels has been reported in humans infected with *P*. *falciparum* prior to the onset of blood-stage infection [25,26]. The blood stage infection, also known as the symptomatic phase, begins with the release of merozoites into the bloodstream and the invasion of RBCs by these merozoites. iRBCs express parasitic antigens (pathogen-associated molecular patterns or PAMPs), which interact with pattern recognition receptors (PRRs) expressed by the innate immune cells like macrophages and dendritic cells. Interaction of PAMPs with receptors such as Toll-like receptor can result in expression of proinflammatory cytokines such as IL1-alpha, IL1-beta, IL6, and TNF [27]. Similarly, glycosylphosphatidylinositols (GPIs) of parasitic origin can induce the expression of TNF in macrophages in vitro [28]. Hemozoin, another PAMP associated with *Plasmodium* infection, induces the macrophages and monocytes to express TNF and IL-1 beta in vitro [29]. iRBC-derived microvesicles have been from shown to stimulate monocyte-derived human macrophages. Upon stimulation, these cells secrete IL-10 and TNF [30]. In line with these reports, TNF, IL1-alpha, and IFN gamma levels are up-regulated in patients with malaria [26,31]. Such systemic inflammation can up-regulate the expression of ICAM1 and VCAM receptors on endothelial cells [32]. This might increase the sequestration of iRBCs on the endothelial cells, further eliciting a proinflammatory response from the endothelial cells. These events can lead up to the development of a proinflammatory microenvironment in the brain vasculature. The chemokines secreted by the endothelial cells can recruit leukocytes such as neutrophils, basophils, and natural killer cells to the site of inflammation. Neutrophils secrete chemokines like MIP-1α, MIP-1β, monocyte chemotactic protein 1 (MCP-1), and IL-8 [24]. Basophils are known to secrete MIP-1α, IL-4, and IL-13 [24]. The chemokines secreted by these cells lead to the migration of more leukocytes to the site of inflammation, thus generating a feed-forward loop of localized inflammation around the vasculature. This, along with the up-regulation of the procoagulation pathway, can affect the functional and structural integrity of brain microvasculature.

### d) *Endothelial breakdown*

The BBB regulates the movement of ions, biomolecules, and other cargo between the brain and the periphery. Disruption of BBB is associated with CM pathology, as evidenced by the extravasation of impermeable dyes into the mice brains in ECM and the presence of haemorrhages in the brains of CM patients [33]. The tight junction proteins such as occludin, vinculin, and zonula occludens 1 (ZO-1) have been shown to be reorganized in the brain sections obtained from fatal human CM cases [34,35]. In vitro studies have shown that interaction between VCAM-1 and PfEMP-1 activates the Rac family small GTPase 1 (Rac1) signalling, which can induce the reorganisation of tight junction proteins [36]. Up-regulation of host microRNA 155 (miR-155) and parasite microRNA miR-451a has also been associated with the reorganisation of one of the proteins present at the tight junction called claudin 1 [37]. Both TNF and IFN-γ are known to up-regulate the levels of miR-155 [37]. Therefore, elevated levels of proinflammatory cytokines can lead to impaired functioning of tight junction proteins. Interaction of PfEMP1 with ICAM-1 leads to phosphorylation of cytoskeletal proteins such as focal adhesion kinase 1 (FAK), paxillin, p130Cas, and cortactin, which can trigger cytoskeletal remodelling of endothelial cells, thus affecting their permeability [38].

Another significant player in the breakdown of BBB is the CD8+ T cells. It has previously been reported that the MHC Class I molecules on the endothelial cells can present parasitic antigens [39]. A widely supported hypothesis was that the parasitic antigen presented by the endothelial cells was recognised by the CD8+ T cell receptor, which led to granzyme B–mediated death of the endothelial cell. Riggle and colleagues, in their seminal work, reported the presence of CD3+ CD8+ T cells in the postmortem brain samples of paediatric CM patients [40]. They also showed that these cells and granzyme B colocalised with the endothelial cells, suggesting that CD8+ T cell–mediated cytotoxicity is involved in CM pathogenesis.

### ii) Pathogenesis in brain parenchyma: Astrocytes, microglia, pericytes, and neurons

The structural disintegration of BBB exacerbates inflammation in the brain. This is mediated by the resident brain cells, astrocytes, and microglia. Astrocytes are glial cells and provide biochemical support to the endothelial cells of BBB. They also help in the regulation of cerebral blood flow. Physical contact with astrocytes increases the expression of proteins associated with the tight junctions in the BBB.

Interestingly, astrocytes in the brains of mice with experimental CM retract their processes from the BBB [41]. This could further weaken and damage the BBB. Astrocytes are also known to secrete chemokine CXCL9, which recruits cytotoxic CD8^+^ T cells to the site of inflammation. In vitro studies have shown that upon stimulation with iRBCs, astrocytes up-regulate the secretion of chemokines such as CCL2 and CCL5 [42]. These chemokines can recruit leukocytes to the sequestration site and aggravate local inflammation.

Microglia are immune cells of hematopoietic origin that reside in the brain. Microglia migrate to the site of any infection or neuronal injury in response to the chemokines and cytokines released at the site and are known to secrete cytokines like TNF and IL-1 beta themselves. The expression of proteins present in the tight junctions like claudin-5, zona occludens-1, occludin, and p-glycoprotein can be regulated by IL-1β and TNF [43]. Both TNF and IL-1β are up-regulated in the brains of CM patients [34]. Microglial activation and dysregulation of the pro- and anti-inflammatory processes in the brain contribute to neuronal dysfunction associated with the CM pathology.

Pericytes are mural cells that wrap around the endothelial cells. They help regulate blood flow in cerebral vasculature and clearance of debris from the neurovascular unit. They induce the expression of tight junction proteins associated with BBB. Pericytes also express angiopoietins. Angiopoietin-1 demonstrates BBB protective effects, whereas angiopoietin-2 destabilises the BBB by weakening endothelial and supporting cells’ interaction [44]. In line with this, angiopoietin-2 expression is up-regulated, and angiopoietin-1 expression is down-regulated in patients with CM [45]. Postmortem studies of CM patients reveal axonal damage and myelin loss in the cerebrum, cerebellum, and brainstem. It has been shown in the mouse model of CM that CD8^+^ T cells secrete granzyme B, which mediates neuronal death via activation of neuronal caspase-3 and calpain-1 [46]. Calpain 1 is associated with actin dynamics and might be responsible for the axonal damage seen in CM patients [47].

## Current intervention strategies

The current recommendation for treatment of severe malaria is to start with a full dose of effective parenteral (or rectal) antimalarial treatment for a minimum period of 24 hours followed by a full dose of effective ACT orally [48]. Anticonvulsants such as phenobarbitone (3.5 mg/kg) reduce the incidence of convulsions but have been shown to be associated with increased mortality in paediatric CM because of respiratory arrest. Hence, WHO recommends that a 20-mg/kg b.w. of phenobarbital should not be given without respiratory support. There is no effective ancillary treatment [49]. Clinical trials with monoclonal anti-TNF antibodies, steroids, and desferrioxamine proved largely unsuccessful. But recent discoveries associated with CM pathogenesis have opened the doors for discussion on several novel therapeutic strategies. For example, a study reported that anti-PfEMP-1 antibodies confer immunity against mild malaria [50]. But the substantial antigenic diversity of PfEMP1is the major hindrance to developing this antigen as a vaccine against *P*. *falciparum* malaria. Two approaches have been suggested to overcome the challenge posed by antigenic diversity: developing a multivalent vaccine (such as the one developed against *Streptococcus pneumoniae*) and targeting conserved epitopes on PfEMP1 [51]. Comprehensive studies to determine the evolutionary diversity of PfEMP1, its spread across different populations, conservation of epitopes, and structural properties will have to be done to support the development of PfEMP1 vaccines. Administration of activated protein C and depletion of lymphotoxin α has demonstrated therapeutic effects by protecting BBB integrity in mouse models of CM [52,53]. Another study reported that treatment with neuregulin-1 reduces the levels of proinflammatory cytokines and decreases leukocyte accumulation in the brains of mice with experimental CM [54]. Depletion of microRNA lowers endothelial activation and reduces vascular leakage in an ex vivo endothelial model [37]. Endothelial dysfunction is associated with low levels of nitric oxide. Supplementation with L-arginine, the precursor of nitric oxide, increases cerebral blood flow and dilation of blood vessels in an experimental model of CM [55]. Even though the studies mentioned above demonstrated promising results, translating them into therapeutic strategies for CM in humans will be challenging.

## Concluding remarks

WHO estimated the global burden of malaria as 241 million cases in 2020 [56]. The total number of deaths due to malaria was estimated to be 0.6 million cases, the highest since 2011 [56]. Global epidemiological data are not available for severe or cerebral malaria. But it has been reported that approximately 1% of the total Pf infections progress to CM [5]. The case fatality rate for CM can be as high as 19% in children and up to 50% in adults [57]. Many patients, both children and adults, who survive CM experience long-term effects such as motor and speech disabilities, cognitive deficits, epilepsy, and neuropsychiatric abnormalities. Identifying new therapeutic targets and agents is essential to reducing the number of deaths due to CM [8]. Better treatment strategies have to be designed to lower the occurrence of long-term sequelae.

The recent discoveries associated with CM pathogenesis further highlight the fact that CM manifests as a complex neurological syndrome. The symptoms and pathological changes in the brain are heterogenous in nature, and a deep understanding of these mechanisms is crucial to develop better intervention strategies. Understanding the role of host and parasite genetics in the degree of severity of malaria, understanding how the host and parasite factors differ between high transmission and low transmission areas, and developing animal models for CM that better mimic the human CM will further our understanding.
